# Memory window engineering of Ta_2_O_5−x_ oxide-based resistive switches via incorporation of various insulating frames

**DOI:** 10.1038/srep30333

**Published:** 2016-07-25

**Authors:** Ah Rahm Lee, Gwang Ho Baek, Tae Yoon Kim, Won Bae Ko, Seung Mo Yang, Jongmin Kim, Hyun Sik Im, Jin Pyo Hong

**Affiliations:** 1 Division of Nanoscale Semiconductor Engineering, Hanyang University, Seoul, 133-791, South Korea; 2 Novel Functional Materials and Devices Lab, The Research Institute for Natural Science, Department of Physics, Hanyang University, Seoul 133-791, South Korea; 3 Department of Semiconductor Science, Dongguk University, Seoul, 100-715, South Korea

## Abstract

Three-dimensional (3D) stackable memory frames, including nano-scaled crossbar arrays, are one of the most reliable building blocks to meet the demand of high-density non-volatile memory electronics. However, their utilization has the disadvantage of introducing issues related to sneak paths, which can negatively impact device performance. We address the enhancement of complementary resistive switching (CRS) features via the incorporation of insulating frames as a generic approach to extend their use; here, a Pt/Ta_2_O_5−x_/Ta/Ta_2_O_5−x_/Pt frame is chosen as the basic CRS cell. The incorporation of Ta/Ta_2_O_5−x_/Ta or Pt/amorphous TaN/Pt insulting frames into the basic CRS cell ensures the appreciably advanced memory features of CRS cells including higher on/off ratios, improved read margins, and increased selectivity without reliability degradation. Experimental observations identified that a suitable insulating frame is crucial for adjusting the abrupt reset events of the switching element, thereby facilitating the enhanced electrical characteristics of CRS cells that are suitable for practical applications.

Resistive random access memory (ReRAM) devices are rapidly becoming one of the most reliable candidates for next-generation emerging memories due to their outstanding features, including their ultra-low power consumption, fast operation, and outstanding scaling potential^1^,^2^,^3^. To ensure such features, a variety of non-stoichiometric transition metal oxide materials have been employed^4^,^5^. Among the materials that have been considered recently, tantalum oxides have been the focus of immense interest as promising candidates for main data storage media due to their fast switching speed on the sub-nanosecond level and their affordable endurance properties of more than 10^12^ cycles^6^,^7^. The widespread use of these materials is likely linked to the formation of simple structural phases, including conductive Ta(O) and insulating Ta_2_O_5_ phases, and their controllable compositions^8^,^9^. The underlying nature of resistive switching (RS) behavior can be described by adapting a filamentary model, such as electrochemical metallization (ECM) phenomena or the valence change mechanism (VCM)^4^,^10^, however, fully understanding the nature of RS behavior remains a challenge.

A simple metal-insulator-metal (MIM) configuration is expected to enable the ongoing miniaturization of memory devices; this should also allow devices to be used in scalable crossbar arrays with an ideal 4F^2^ memory cell size^11^,^12^. However, attractive crossbar array configurations, which are crucial for the realization of high density ReRAM devices, are highly susceptible to sneak path issues that arise from undesired leakage currents through neighboring cells^12^,^13^,^14^. Therefore, the use of a selection device, such as a diode or transistor, is required to overcome these problems at each cross-point^4^,^14^. Recently, Waser’s group established a complementary resistive switching (CRS) concept that has garnered considerable interest as a reliable option to overcome sneak path issues without the use of a selection device^15^. The CRS cell consists of two anti-serially merged bipolar switching elements that share a common middle electrode. The operation of CRS cells depend on either the ECM or VCM behavior^16^,^17^. The superimposed I–V response showed typical CRS behavior, demonstrating the potential to suppress sneak path currents without using selection devices. To date, numerous reports have been published that successfully demonstrate ECM-based CRS devices and their applications in various crossbar array architectures^17^,^18^,^19^. Several groups suggest alternative operating schemes and confirm the further improved features, which provide the highly reduced high noise margin sneak path current in a crossbar array structure^20^,^21^,^22^. In addition, an associative capacitive network based on CRS, which can be used as a memory-intensive platform with a significant area and energy efficiency, was introduced in the 40 X 41 nano-crossbar array^23^. Beyond their use in non-volatile memory applications, CRS devices have recently been used in a variety of devices, such as multi-functional memories^24^, neuromorphic computing^17^, and fuzzy logic circuits^19^. However, further studies are necessary for VCM-based CRS cells that adapt a fully-stacked M/I/M/I/M structure; this is the case because VCM-based CRS cells have exhibited several key issues affecting device performance, such as a low on/off ratio, ON-state degradation, small read margins, etc. In addition, the symmetric SET and RESET threshold voltages for CRS require the use of a suitable resistor in series in order to establish a functional memory margin. Therefore, manipulation of the aforementioned issues represents a key requirement to enable the development of various VCM-based CRS devices.

In this letter, we introduce a generic approach for advancing VCM-based CRS characteristics by incorporating insulating frames into a fully-stacked Pt/Ta_2_O_5−x_/Ta/Ta_2_O_5−x_/Pt MIMIM CRS configuration. The primary principle underlying our experiment is to intentionally induce a high voltage reset process after the set process; this is done by including additional insulating frames during the CRS. We first examined the direct serial connection of external variable resistors to a basic CRS or bipolar resistive switching (BRS) device to verify the functionality of the additional resistor on the electrical performance of CRS or bipolar resistive switching (BRS) devices. Secondly, the insertion of single or double Ta/Ta_2_O_5−x_/Ta insulating frames into the middle Ta electrode of the CRS device was tested and the structural features were analyzed. Furthermore, a Pt/a-TaN/Pt/Ta_2_O_5−x_/Ta/Ta_2_O_5−x_/Pt structure, containing an amorphous a-TaN insulating layer, was also employed to simplify the overall CRS configuration. These experimental findings can be generalized and extended to engineer CRS switching characteristics that are suitable for high-density CRS application in array-level structures.

## Results and Discussion

**Figure 1:**
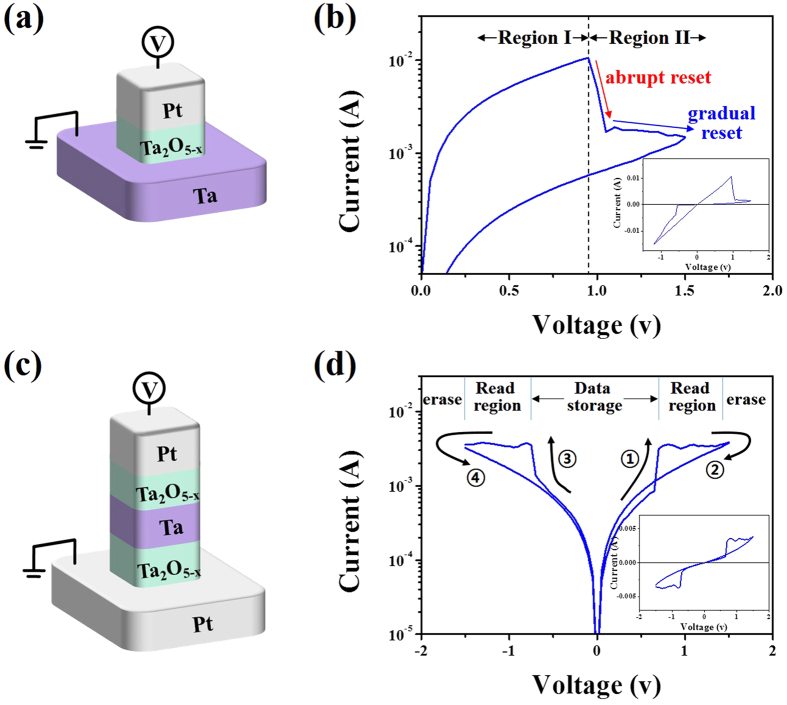
Schematics and corresponding I–V characteristics of typical BRS and CRS cells. (**a**) Schematic and (**b**) typical I–V response of a Pt/Ta_2_O_5−x_/Ta bipolar switching cell; the application of a voltage clearly causes abrupt and gradual reset events in the positive sweeping region. The inset shows the same I–V plot recorded on a linear scale for the whole voltage sweep region. (**c**) Schematic and (**d**) typical I–V response of the Pt/Ta_2_O_5−x_/Ta/Ta_2_O_5−x_/Pt CRS cell; the inset is the same plot acquired on a linear scale.

Figure 1a,b plot the schematics and representative I–V response of a typical Pt/Ta_2_O_5−x_/Ta bipolar switching element, respectively. The resistive switching behavior during the reset process and the inset exhibiting the same I–V response on a logarithmic scale for the whole bias region are highlighted. The observed bipolar resistive switching behavior can be understood based on the filamentary model; this is caused by the reorganization of oxygen vacancies in the Ta_2_O_5−x_ layer^9^,^25^,^26^. As seen in this figure, the reset process can be divided into two regions describing abrupt and gradual reset behaviors: Region I and Region II. We expect that Region I corresponds to the ON-state provided by a metallic filament, while Region II is likely associated with a gradual dissolution process of the residual filaments after the rupture of the main filament path^27^,^28^. The reset voltage-dependent behaviors of a typical switching element are discussed in Figure S1 (Supporting Information), identifying the presence of relevant multileveled I–V responses that depend on the reset voltages. However, more detailed investigation into the nature of these two different regions is still needed in order to establish a clearer explanation for the underlying mechanism observed in these samples. Figure 1c exhibits the schematic of Pt/Ta_2_O_5−x_/Ta/Ta_2_O_5−x_/Pt CRS switching cells that are anti-serially connected with two basic bipolar Pt/Ta_2_O_5−x_/Ta switching elements; here, a Ta layer is commonly used as the middle electrode of the CRS cell. For convenience, the Pt/Ta_2_O_5−x_/Ta and Ta/Ta_2_O_5−x_/Pt structures of the CRS cell are referred to as the top and bottom switching elements, respectively. The corresponding representative I–V curve is shown in Fig. 1d. The direction of the applied electric field and classifications according to the functional region are included. The initial forming process and relevant I–V curves of Pt/Ta_2_O_5−x_/Ta/Ta_2_O_5−x_/Pt CRS cell are given in Figure S2 (Supporting Information). The inset of Fig. 1d plots the same I–V curve on a semi-logarithmic scale. The bias voltage is swept in the sequence of 0 V → 1.5 V → 0 V → −1.5 V → 0 V. The cell is switched from the low resistance state (LRS) of the top switching element and the high resistance state (HRS) of the bottom switching element to ‘LRS/LRS’ at step ①. A negative differential resistance region (NDR) was observed immediately, leading the cell to gradually adopt an ‘HRS/LRS’ configuration until reaching step ②. Consequently, a similar trend also occurs in the negative bias region. The cell switches to an ‘LRS/LRS’ configuration at step ③ and returns to the ‘LRS/HRS’ configuration at step ④. As seen in this figure, the CRS cell exhibited a low ON-window with a low on/off ratio and a narrow read region. This is mainly due to the fact that the gradual reset process occurs in Region II (as mentioned in Fig. 1b), right after the set process participates in the CRS operation. Alternatively, Region I is not involved in the CRS due to the appearance of the relatively similar set and reset voltages in the top and bottom switching elements. In addition, the symmetric set and reset voltages in each switching element cause the read region required for the CRS reading process to become narrower and to provide noticeably lower selectivity, which is defined as S = I @ V_read_/I @ 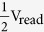. This limits the practical applications. In particular, the CRS behavior frequently disappeared during repeated switching measurements. This is directly the consequence of the relatively unstable gradual reset process in Region II of the switching element during CRS operation.

**Figure 2:**
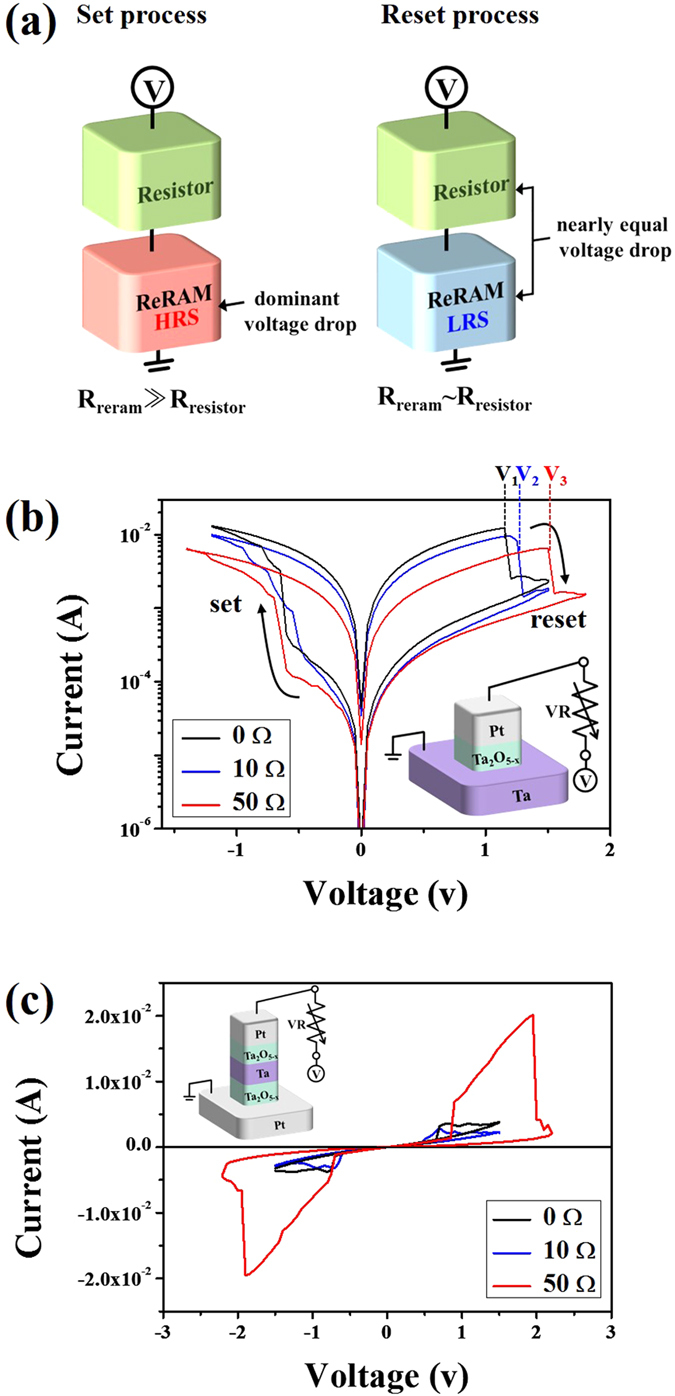
(**a**) External serial resistor (green color)-dependent cell architectures depicting possible voltage drops that occur during the set and reset process. Dominant voltage drop during the set process appears in the switching cell (red color), while approximately comparable voltage drops between the switching cell (blue color) and external resistor (green color) occur during the reset process. The corresponding I–V responses of (**b**) the Pt/Ta_2_O_5−x_/Ta bipolar switching cell and (**c**) the Pt/Ta_2_O_5−x_/Ta/Ta_2_O_5−x_/Pt CRS cell serially connected with various external resistors.

In an attempt to solve the aforementioned issues, we first adopt a direct connection between an external resistor and a bipolar switching element to shift the reset voltage to a higher voltage region. Achieving a higher reset voltage is expected to allow for the use of a relatively higher ON-current, which occurs in Region I of the switching element during CRS operation. Figure 2a shows the schematic of the bipolar switching element serially connected to an external resistor with several tens of ohms. The typical bipolar switching element provides several gigaohms in the HRS and several tens of ohms (approx. 90 Ω) in the LRS. Thus, the HRS resistance of the switching element is much higher than that of the external resistor. During the set process, the dominant voltage drops in the HRS state of the switching element, while the set voltage of the switching element remains mostly unaffected. However, when the switching element is in the LRS during the reset process, the LRS resistance of the switching element is comparable to that of the external resistor. This means that the voltage drops in the external resistor and the switching element are comparable, which affects the reset voltage of the switching element. In order to gain insight into how incorporating an external resistor influences the BRS characteristics, commercially-available resistors in the range of 10 and 50 Ω were electrically connected to the Pt/Ta_2_O_5−x_/Ta sample. Various external resistors (comparable with the LRS resistances of the switching element) were chosen to initiate proper BRS events. The corresponding results are plotted in Fig. 2b. As expected, the incorporation of serially connected resistors contributes to a reset voltage shift in the switching elements (denoted as V_1_, V_2_, and V_3_ in the graph) as well as a slightly decreased ON-current level. However, the set voltages remain mostly unchanged. Similar analyses were carried out on a basic Pt/Ta_2_O_5−x_/Ta/Ta_2_O_5−x_/Pt CRS cell by adopting serially connected external resistors. The CRS cells also verified the presence of delayed abrupt reset events followed by a set event. In particular, the use of a 50 Ω external resistor demonstrates the enhanced memory margins. In general, increasing the external resistance in a BRS cell leads to slightly decreased ON-current levels (red line); this is due to the increasing voltage drop over the external serial resistor^17^, as illustrated in Fig. 2b. However, incorporating the external resistor causes the asymmetric set and reset voltages (V_reset_ > V_set_) of the BRS elements to increase. This allowed the CRS cell to utilize a high ON-current, which initially occurred in Region I of the BRS cell. Thus, the CRS cell enabled an enlarged ON memory window with a higher ON-current (red line), as seen in Fig. 2c.

**Figure 3:**
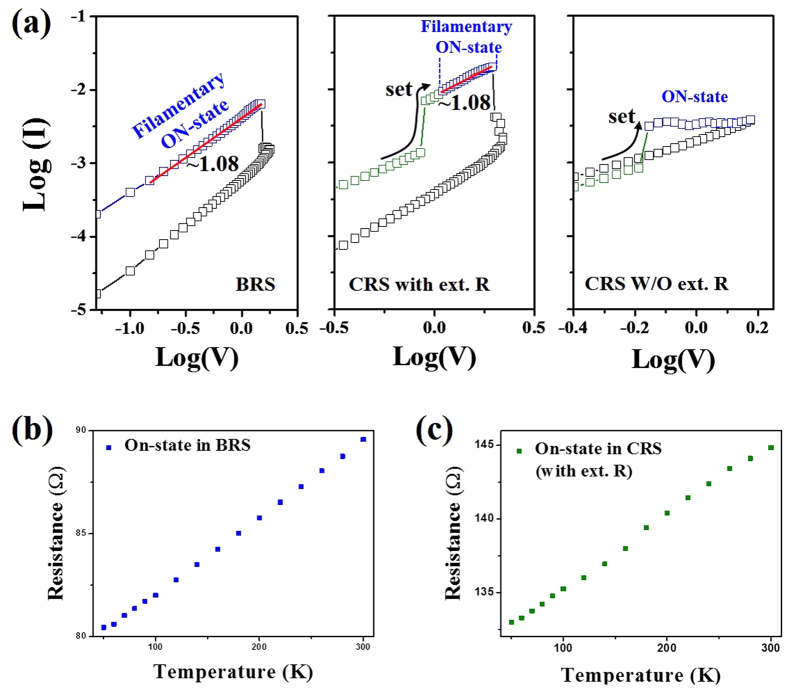
(**a**) Log-log scale I–V characteristics of bipolar (left), CRS cell integrated with an external 50 Ω resistor (middle), and CRS cell without an external resistor (right) in the positive region. Temperature-dependent resistances in the ON-states of (**b**) BRS and (**c**) CRS cells with a 50 Ω resistor between 50 and 300 K.

To identify the conduction nature of ON-states, the I–V curves of BRS and CRS cells were re-plotted on a log-log scale, as shown in Fig. 3a. The ON-state slope (blue dots in the left panel of Fig. 3a) of BRS was approximately equal to 1 (1.08), reflecting the creation of conductive filaments in the BRS cell^29^. The slope is well-described by Ohm’s law. In addition, an approximately similar slope of 1.08 was also observed for the ON-state (blue dots in the middle panel of Fig. 3a) of the CRS cell after completion of the set process (green dots), while that of CRS without an external resistor was obtained much of the different value (blue dots in the right panel of Fig. 3a). The similar slopes suggest that the ON-current of the CRS cell with an external resistor was caused by the presence of completely formed filaments in the CRS cell, unlike that of the typical CRS without an additional resistor. To further validate the nature of the electrical transport regarding the ON-states, temperature-dependent resistances of the ON-states for BRS and CRS with an external resistance were also recorded. These data were taken at 0.2 V. As shown in Fig. 3b,c, the resistances for both samples increased with increasing temperatures, reflecting the metallic-like behavior that is caused by the completely grown conducting filaments.

**Figure 4:**
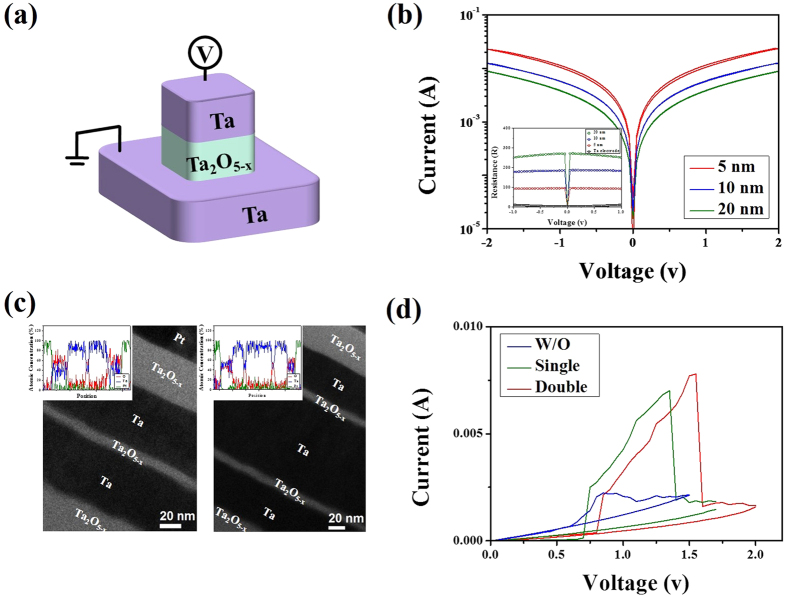
(**a**) Sketch of a Ta/Ta_2_O_5−x_/Ta frame working as an additional resistor. (**b**) Ta_2_O_5−x_ thickness-dependent I–V curves of Ta/Ta_2_O_5−x_/Ta frames. Note that the inset reveals the Ta_2_O_5−x_ thickness-dependent resistance values. (**c**) Cross-sectional TEM image of fully-stacked Pt/Ta_2_O_5−x_/[Ta/Ta_2_O_5−x_/Ta]/Ta_2_O_5−x_/Pt and Pt/Ta_2_O_5−x_/[Ta/Ta_2_O_5−x_/Ta/Ta_2_O_5−x_/Ta]/Ta_2_O_5−x_/Pt CRS cells. The insets show the relevant EDS elemental line scan results. (**d**) Representative I–V responses of CRS cells serially connected without (blue line) and with the inserted single (green line) or double (red line) Ta/Ta_2_O_5−x_/Ta frames. “Single” and “double” refer to Ta/Ta_2_O_5−x_/Ta and Ta/Ta_2_O_5−x_/Ta/Ta_2_O_5−x_/Ta frame insertions in the CRS cell, respectively.

To further clarify the CRS characteristics regarding the effect of additional resistance, a symmetric Ta/Ta_2_O_5−x_/Ta frame (acting as an additional resistor in the CRS cell) was prepared, as shown in Fig. 4a. One striking feature of this device is the absence of obvious RS behavior within ±2 V in the Ta/Ta_2_O_5−x_/Ta frame. Thus, all frames show nearly linear I–V curves with a monotonic decrease in the current levels that depends on the increasing Ta_2_O_5−x_ thickness. In particular, a 10-nm-thick Ta_2_O_5−x_ frame exhibited a nearly constant resistance (approx. 170 Ω) without any hysteresis during voltage sweeping, as shown in the inset of Fig. 4b. This indicates that a proper Ta/Ta_2_O_5−x_/Ta frame can be used as an additional resistor in our CRS system. Therefore, to demonstrate the role of the designed additional resistor, single and double Ta/10-nm-thick Ta_2_O_5−x_/Ta frames were inserted into the middle electrode of basic Pt/Ta_2_O_5−x_/Ta/Ta_2_O_5−x_/Pt CRS frames. Two stack architectures were prepared: Pt/Ta_2_O_5−x_/[Ta/Ta_2_O_5−x_/Ta]/Ta_2_O_5−x_/Pt (Sample A) and Pt/Ta_2_O_5−x_/[Ta/Ta_2_O_5−x_/Ta/Ta_2_O_5−x_/Ta]/Ta_2_O_5−x_/Pt (Sample B). Samples A and B denote single and double insertions of Ta/Ta_2_O_5−x_/Ta insulting frames into the basic CRS cell, respectively, where the thickness of the Ta_2_O_5−x_ layer in the top and bottom bipolar switching elements was 50 nm. Figure 4c shows a typical cross-sectional TEM image of Samples A (left image) and B (right image), exhibiting multiple stacked configurations with clear interfaces. The insets of each TEM image show the energy-dispersive X-ray spectroscopy (EDS) line profiles containing the atomic percentages, which are in good agreement with the TEM image. Figure 4d plots the CRS features of Samples A and B. As seen in this figure, the I–V responses of Samples A and B exhibited similar trends with those of Fig. 2. Samples A and B maintained sufficient ON-states until reaching abrupt reset events at 1.35 V and 1.6 V after the set events, respectively. These experimental findings verified the enlargement of the ON-windows (green and red lines) and the read region in CRS cells that are caused by incorporating additional insulating frames. To further validate their use regarding the above observations, an amorphous tantalum nitride (a-TaN) material was also examined as a potential resistor since it is believed that the TaN is a valuable material working as one of resistors due to low temperature coefficient of resistivity in electronic device and chemically inert features^30^,^31^. Further detailed characterizing of TaN films prepared by reactive sputtering of a Ta Target in N_2_ + Ar gas mixtures is discussed in Figure S3 (Supplementary Information). Additionally, the composition of a-TaN was Ta_1_N_1.26_ was taken from the Rutherford backscattering spectroscopy (RBS) measurements.

**Figure 5:**
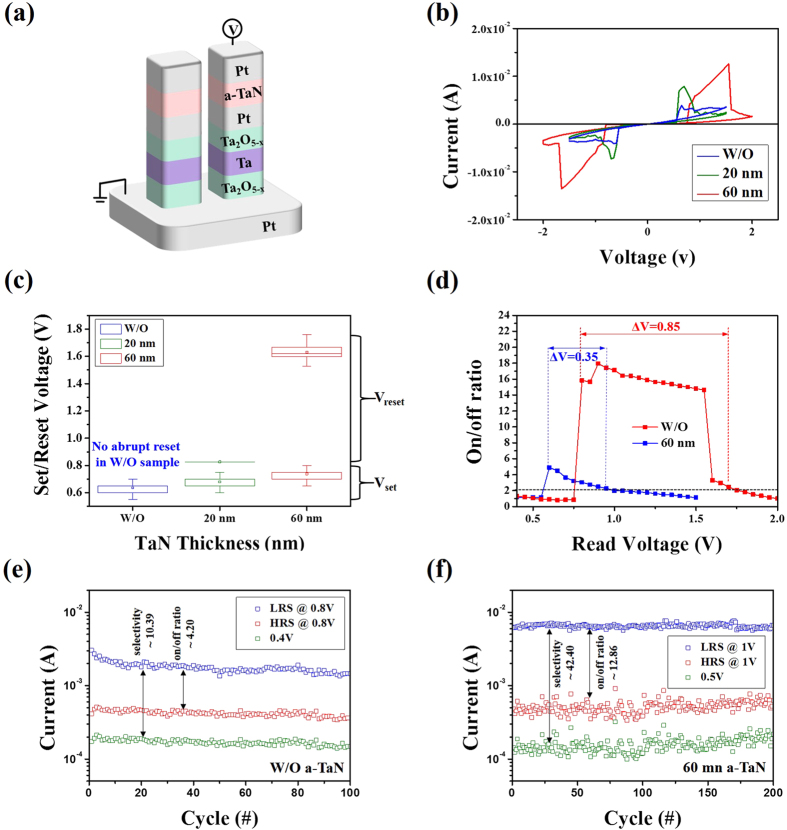
Effect of an additional a-TaN layer on the I–V features of the CRS cell. (**a**) Schematic of the CRS device serially connected with the a-TaN layer. (**b**) Representative I–V responses of the CRS cells, where 20-nm-thick (green line) and 60-nm-thick (red line) a-TaN layers were used for the measurements. (**c**) Statistics for V_set_ and abrupt V_reset_ distributions, depending on the a-TaN layer thickness. (**d**) On/off ratios obtained in the positive voltage region, yielding clear evidence of the enhanced read margins caused by the a-TaN layer. Endurance features of CRS cells (**e**) without and (**f**) with the 60-nm-thick a-TaN layer.

Figure 5a shows the schematic of a basic CRS cell integrated with a Pt/a-TaN/Pt insulting frame (Sample C series). Figure 5b plots the corresponding I–V responses of the Sample C series along with the results of the basic CRS cell (without an additional insulating frame). The thicknesses of the Sample C series were 20 and 60 nm. As seen in this figure, enlarged memory windows (green and red lines) were observed, along with increased ON-current levels. Figure 5c shows the distribution of the set and reset voltages for all samples in the positive region taken from 100 relevant consecutive switches. In the set and reset voltage measurements, the reset voltages were selected at the point where the abrupt reset process takes place. However, since the sample without an a-TaN frame did not reveal an abrupt reset process during CRS operation (only showing a gradual reset process), the reset voltage of this sample was not included in Fig. 5c. In other words, the incorporation of an additional a-TaN layer contributes to an increase in the reset voltage of the CRS cell, while also increasing the set voltage slightly. To determine an effective read region (ΔV) where the on/off ratio is two times higher, the on/off ratios of CRS cells without and with a-60-nm thick a-TaN layer are evaluated in the positive bias region, as shown in Fig. 5d. The CRS cell with a 60-nm-thick a-TaN layer reveals a significantly larger ΔV than that of the basic CRS cell (0.35 V → 0.85 V). This notably higher on/off ratio implies the enhanced effective read region that is created by using a suitable a-TaN layer. Figure 5e,f show the reliable endurance features of CRS cells without and with a 60-nm-thick a-TaN layer operated for 100 continuous dc switching cycles, respectively. Both cells displayed stable on/off ratios at V_read_ and self-selective behavior at a 1/2 V_read_ without significant current degradation. As shown in this figure, the sample with the 60-nm-thick a-TaN provided a higher mean on/off ratio (4.2 → 12.86) and selectivity (10.39 → 42.40) compared to the sample without an a-TaN layer. Thus, the observed enhancement in device performance is caused by the use of a suitable insulating frame.

But, the incorporation of insulating frames into the CRS structure that we have addressed in this work also has its own limitations. At first, the operation voltage increases with increasing resistance of series resistors. Especially, since the set voltage is insensitive to the series resistor, the reset voltage arising from the series resistor could be increased, possibly leading to other issues on neighbor cells in the array structure during the erase process. Secondly, the use of proper additional resistors can create the enlargement in ON-current and selectivity only until the VCM-based CRS utilizes the filamentary-assisted Region I. However, if series resistance is further increased, the maximum ON current would be decreased due to the increased voltage drop over the series resistance. This would be the same to the results observed by typical CBM-based CRS device^17^. Therefore, one of the crucial steps towards solving the above issues will be the choice of serial resistor with proper resistance to satisfy essential conditions of single device when applied to the array structures.

## Conclusion

In summary, the incorporation of various insulating frames into the basic CRS structure was systematically examined in an attempt to enhance the oxide-based CRS characteristics. Experimental findings provided evidence that using a suitable additional resistor layer can enlarge the read margin and on/off ratio and improve the selectivity. This behavior is explained by the presence of a voltage drop in the additional resistor, which causes the reset process to occur at a higher voltage. The delayed reset voltage allows for the use of a high ON-current region, which is induced by the conductive filament in the essential bipolar switching element of the CRS device. Two insulating configurations (Ta/Ta_2_O_5−x_/Ta and amorphous TaN) serve as additional resistors and clearly lead to enhanced device performance. The read region, on/off ratio, and selectivity are all improved compared to the basic CRS device. Thus, we anticipate that the ability to improve the electrical features of CRS devices by properly including an insulating frame will lead to practical applications for valence change-based CRS systems.

## Methods

Various stack structures were prepared as follows: Pt/Ta_2_O_5−x_/Ta for BRS cells and Pt/Ta_2_O_5−x_/Ta/Ta_2_O_5−x_/Pt, Pt/Ta_2_O_5−x_/Ta/Ta_2_O_5−x_/Ta/Ta_2_O_5−x_/Pt, Pt/Ta_2_O_5−x_/Ta/Ta_2_O_5−x_/Ta/Ta_2_O_5−x_/Ta/Ta_2_O_5−x_/Pt, and Pt/a-TaN/Pt/Ta_2_O_5−x_/Ta/Ta_2_O_5−x_/Pt for CRS cells including a Ta/Ta_2_O_5−x_/Ta insulating frame. All samples were fabricated on SiO_2_/Si substrates using a sputtering system and each Pt, Ta, and Ta_2_O_5−x_ layer was deposited using the same fabrication conditions. For the Pt/Ta_2_O_5−x_/Ta bipolar switching element shown in Fig. 1a, a 100-nm-thick Ta bottom electrode was grown by an RF magnetron sputtering system. 100-μm-square patterns were subsequently deposited with a conventional photolithography method. Next, an amorphous, 50-nm-thick Ta_2_O_5−x_ layer was deposited on the Ta bottom electrode as an active layer using an RF sputtering technique at room temperature where a Ta_2_O_5_ ceramic target was employed under pure Ar ambient. Detail physical properties of Ta_2_O_5−x_ thin film are given in Figure 4S (Supplementary Information). This was followed by the deposition of a 100-nm-thick Pt top electrode on the Ta_2_O_5−x_ layer. For a basic CRS cell, as shown in Fig. 1c, a Pt/Ta_2_O_5−x_/Ta/Ta_2_O_5−x_ configuration was deposited on the bottom Pt electrode using the above process; this was followed by a lift-off photolithographic process. All other samples were prepared in a similar fashion for comparison. In order to evaluate the properties of the TaN film as an insulator layer, the N_2_ flow rate was varied from 0 sccm to 10 sccm with a fixed Ar flow rate (including a N_2_-only condition (10 sccm)) to produce different nitrogen contents in the TaN film during the sputtering process by using a pure Ta target. Electrical analyses were carried out with a Keithley 4200 semiconductor parameter analyzer at RT. A voltage was applied to the top electrode and the bottom electrode was grounded during measurements. Cross-sectional observations of the Pt/Ta_2_O_5−x_/Ta/Ta_2_O_5−x_/Ta/Ta_2_O_5−x_/Pt and Pt/Ta_2_O_5−x_/Ta/Ta_2_O_5−x_/Ta/Ta_2_O_5−x_/Ta/Ta_2_O_5−x_/Pt structures were taken via transmission electron microscopy (TEM). X-ray diffraction (XRD) and the four-probe method were utilized to investigate the properties of the TaN thin film.

## Additional Information

**How to cite this article**: Lee, A. R. *et al.* Memory window engineering of Ta_2_O_5−x_ oxide-based resistive switches via incorporation of various insulating frames. *Sci. Rep.* **6**, 30333; doi: 10.1038/srep30333 (2016).

## Supplementary Material

Supplementary Information
