# Supplementary material for: Memory window engineering of Ta2O5−x oxide-based resistive switches via incorporation of various insulating frames
Source: Sci Rep. 2016 Jul 25;6:30333. doi: 10.1038/srep30333 (PMC4958974; doi:10.1038/srep30333)
Supplement: Supplementary Information [file srep30333-s1.pdf]

SUPPLEMENTARY INFORMATION

Correspondence and requests for materials should be addressed to J.H (jphong@hanyang.ac.kr)

Memory window engineering of Ta_2O_{5-x} oxide-based resistive switches via incorporation of various insulating frames

Ah Rahm Lee, Gwang Ho Baek, Tae Yoon Kim, Won Bae Ko, Seung Mo Yang, Jongmin Kim, Hyun Sik Im, and Jin Pyo Hong*

1. Reset-stop voltage contributions to electrical responses of basic $Pt/Ta_2O_{5-x}/Ta$ switching element

It is believed that the switching nature of our basic switching element $Pt/Ta_2O_{5-x}/Ta$ is expected to originate from a redox process acting upon the filament paths due to the bias-driven drift of oxygen ions in our work. Thus, manipulation of the extent of filament oxidation by adjusting reset-stop voltages would lead to the presence of multistate resistances in the HRS during a reset process, as shown in the figure below (left). As seen in this figure, the current levels of HRS strongly depend on reset voltages. Thus, the HRS current level of single CRS cells can be controlled in our work. Typical I-V features observed for a $Pt/Ta_2O_{5-x}/Ta$ device at various reset-stop voltages ranging from 1.3 to 2.2 V confirmed this hypothesis; apparent various resistances were induced, identifying the clear presence of multi-current levels, as shown in Figures S1.

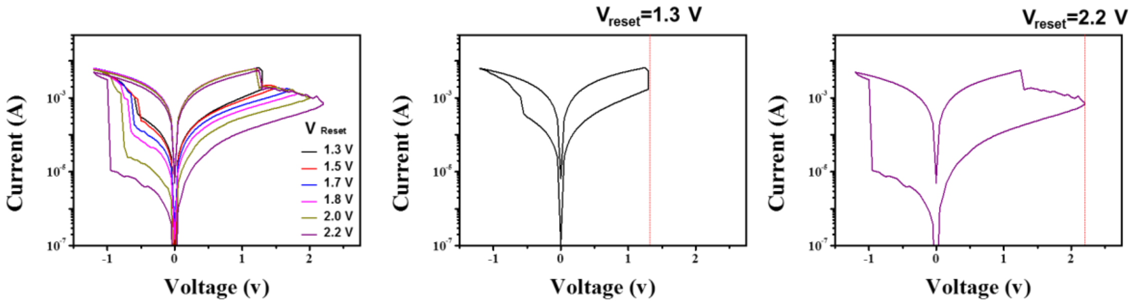

Figure S1 I–V responses of the basic $Pt/Ta_2O_{5-x}/Ta$ switching element observed for various reset-stop voltages.

2. Electrical I-V characteristics of Pt/Ta_2O_{5-x}/Ta/Ta_2O_{5-x}/Pt cell during forming process

Basically, a two-step electroforming process is required to induce CRS features in our configurations, as shown in figure S2. In the initial state (as-grown), the top and bottom Ta_2O_{5-x} layers (switching elements) are in a high resistance state (HRS). Therefore, the fully stacked structure initially remains in a HRS (top)/HRS (bottom) state and acts as a voltage divider under a bias. Ideally, the top and bottom Ta_2O_{5-x} layers, which have the same thickness, should possess the same resistance corresponding to an equal voltage drop under a bias. However, it seems that the resistance of top and bottom switching elements might be different due to the natural cell variation and limitation of fabrication process. For CRS, if the first forming step (black line) is applied, either of top and bottom switching element which possess smaller resistance than the other will produce the LRS state, while the other switching element will stay in the as-grown state. Thus, the CRS cell will remain in an OFF state. Therefore, the CRS cell requires an additional second forming step (red line) for which to switch to the ON state. During the first and second forming step, the compliance current was set 1 and 10 mA to protect the CRS cell from a permanent breakdown, respectively. It should also be noted that the first and second forming processes take place at opposite polarity in our configurations to obtain a stable and symmetric CRS features due to the polarity dependence of the forming process in bipolar resistive switching. Next, it is followed that reset process without a compliance current was performed for CRS cell to be in HRS/LRS or LRS/HRS, allowing the device to be ready for a repeatable CRS features. Finally, the expected superimposed CRS curves (purple and green lines) are observed between -1.5 V and 1.5 V.

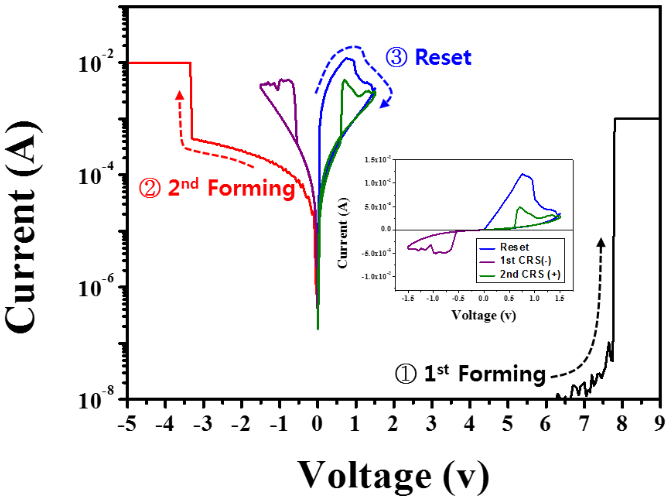

Figure S2 Electric I-V characteristics of a Pt/Ta_2O_{5-x}/Ta/Ta_2O_{5-x}/Pt CRS cell during the forming process, reset, and initial CRS curves. The inset shows the I-V characteristics on a linear scale.

3. Characteristics of TaN films prepared by reactive sputtering process

The TaN thin films were prepared on amorphous SiO_2 substrates at different gas flow conditions. Figure S3a shows the XRD pattern of TaN films as a function of various nitrogen gas flow rates in an Ar + N_2 mixture. At a low N_2 flow rate (0.4 sccm), the (110) peak of the bcc phase was observed. A higher N_2 flow rate (1.6 sccm) indicates the presence of the fcc phase with a preferential orientation along the (111) direction.[26] When exposed to only nitrogen, an amorphous phase was obtained. In addition, the electrical resistivity measurements indicate that the resistivity varies depending on the N_2 flow rate, as shown in Figure S3b. Thus, amorphous TaN (a-TaN), which possesses a higher resistivity, was chosen as an additional resistor in this work.

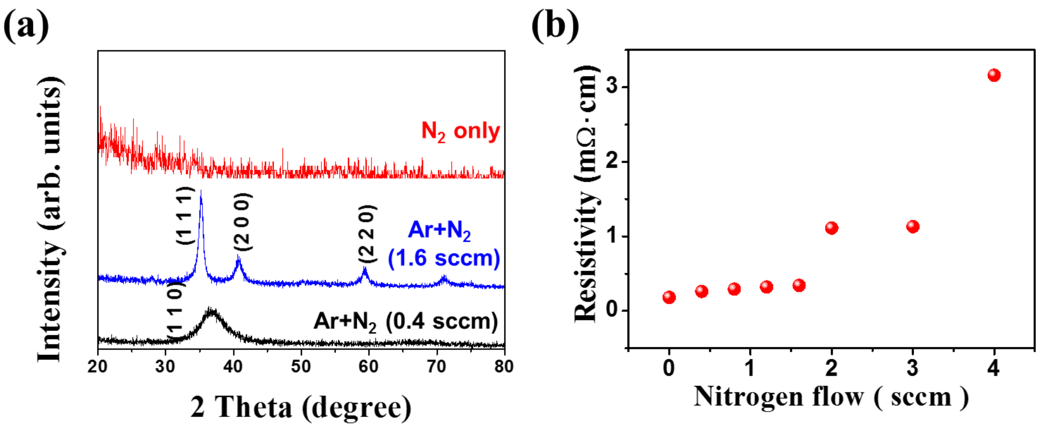

Figure S3 (a) XRD patterns and (b) resistivity plot of the TaN layer as a function of nitrogen gas flow. A relatively resistive TaN layer (amorphous TaN) grown at only 10 sccm nitrogen was chosen to be the additional resistor used for serial connection with the pure BPS and CRS cells.

4. XRD and RBS analyses of Ta_2O_{5-x} thin film

The X-ray diffraction (XRD) patterns of Ta_2O_{5-x} films deposited on SiO_2/Si substrates provided no obvious diffraction peaks, demonstrating the formation of amorphous-phase Ta_2O_{5-x} films (Figure S4a). The RBS spectra of the Ta_2O_{5-x} film (Figure S4b) identified the composition of the films used herein to be $Ta_2O_{4.86}$.

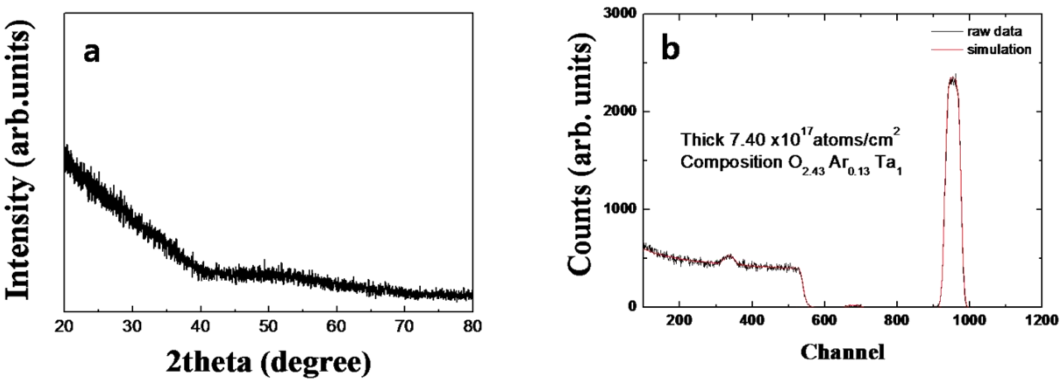

Figure S4. (a) XRD patterns of the Ta_2O_{5-x} layer. (b) RBS profiles of the Ta_2O_{5-x} layer.
